# The Expanding Riboverse

**DOI:** 10.3390/cells8101205

**Published:** 2019-10-05

**Authors:** Sergey O. Sulima, Jonathan D. Dinman

**Affiliations:** 1 Biopharmaceutical New Technologies (BioNTech) Corporation, 55131 Mainz, Germany; sergey.o.sulima@gmail.com; 2 Department of Cell Biology and Molecular Genetics, University of Maryland, College Park, MD 20742, USA

**Keywords:** ribosome, specialization, heterogeneity, cancer, ribosomopathies, translational fidelity

## Abstract

Subverting the conventional concept of “the” ribosome, a wealth of information gleaned from recent studies is revealing a much more diverse and dynamic ribosomal reality than has traditionally been thought possible. A diverse array of researchers is collectively illuminating a universe of heterogeneous and adaptable ribosomes harboring differences in composition and regulatory capacity: These differences enable specialization. The expanding universe of ribosomes not only comprises an incredible richness in ribosomal specialization between species, but also within the same tissues and even cells. In this review, we discuss ribosomal heterogeneity and speculate how the emerging understanding of the ribosomal repertoire is impacting the biological sciences today. Targeting pathogen-specific and pathological “diseased” ribosomes promises to provide new treatment options for patients, and potential applications for “designer ribosomes” are within reach. Our deepening understanding of and ability to manipulate the ribosome are establishing both the technological and theoretical foundations for major advances for the 21st century and beyond.

## 1. Introduction

Contrary to beliefs held by children and certain politicians, the universe does not revolve around ourselves. The Copernican revolution gave rise to a series of profound changes, initiating a shift from a world view dominated by opaque and unknowable supernatural beings to one governed by laws that are accessible to the human mind through the scientific process. In our lifetimes, the discovery of exoplanets, gravitational waves, and multiverse theory is arguably having similar impact. However, the concept of strength through diversity, while a cornerstone of the new cosmology, evolutionary biology and liberal politics, is something that is only emerging in the molecular biosciences. The prevailing view of messenger RNAs (mRNAs) carrying an invariant genetic code that is translated by monochromatic ribosomes to produce identical proteins is only now becoming viewed as simplistic. We are on the cusp of appreciating how diversity at the molecular level may confer selective advantages by broadening both the coding and buffering capacity of the cells and organisms.

When discussing the molecular apparatus that translates genetic information in mRNAs into proteins, our very language betrays the prevailing monolithic view: We tend to refer to “The Ribosome”, as if this complex assemblage of RNAs and proteins is uniform in every living organism. This assumption was not the case in the beginning. After the discovery that genetic information is encoded in nucleic acids and that it is decoded by RNA-rich ribosomes, Francis Crick briefly proposed that each ribosome encoded its own unique protein [1], an idea that was quickly discarded by the discovery of mRNA. A western cultural bias for unifying theories led to the near universal adoption of a single model organism (*Escherichia coli*), while technological limitations necessitated making steady-state measurements in bulk systems: These factors helped to cement the idea of “The Ribosome” in the scientific domain. Even as recently as the turn of the millennium, when atomic resolution structures were first being revealed, the technological reliance on ribosomes that were uniformly packaged into crystals reinforced this monolithic ribosome bias.

A closer look at the literature reveals plentiful contradictory evidence going back as far as the late 1980s. The observation that the interactions between ribosomes and ternary complex were enhanced in isolates from naturally occurring *E. coli* strains, and that this correlated with enhanced growth and survival phenotypes relative to laboratory strains, suggested that ribosomal diversity may have a selective advantage in nature [2]. Another early hint came from findings that deletion of one but not the other ribosomal protein paralogous genes impaired the ability of *Saccharomyces cerevisiae* to propagate the yeast “killer virus” [3]. Later studies using the yeast deletion collection revealed differences in many cellular and metabolic functions [4] and translational accuracy [5], and a recent study revealed that ribosomal protein deletion-specific differences in gene expression signatures were associated with cellular growth rate [6]. Additionally, tissue-specific ribosomal heterogeneity observed both at the level of core ribosomal proteins and regulation suggests that specialized ribosomes naturally exist (reviewed in [7,8]). More recent studies have revealed requirements for specific ribosomal proteins for translation of cellular mRNAs containing internal ribosome entry site (IRES) elements during mouse development [9], and translational regulation in *Drosophila melanogaster* spermatogenesis [10]. Thus, similar to how the discovery of the first exoplanet, 51 Pegasi b, revolutionized our understanding of our place in the universe [11], recent evidence supporting the existence of specialized ribosomes has changed our view of the “riboverse”. Here, we borrow terminology from astronomy and explore this constantly expanding riboverse, and the implications of this growing knowledge on cellular life, industrial applications, and therapeutics.

## 2. Aliens Among Us

Mammalian ribosomes are 80S particles consisting of 80 ribosomal proteins (RPs) and 4 ribosomal RNA (rRNA) molecules in an approximately 1:1 mass ratio. However, other seemingly alien, ”specialized” ribosomes have always thrived in plain sight. Several salient examples are discussed below.

### 2.1. Mitoribosomes and Chlororibosomes

The vast majority of the human proteome is synthesized by cytoplasmic or endoplasmic reticulum-associated ribosomes. However, 13 proteins of the mitochondrial oxidative phosphorylation machinery are translated exclusively by mitochondrial ribosomes (mitoribosomes), assembled from special mitochondrial RPs (encoded by nuclear DNA) and rRNAs encoded in the mitochondrial genome. Mitochondria are generally believed to be descendants of an ancient bacterium that entered into a symbiotic relationship with a primordial single-cell organism. As a result, the 55S mitochondrial ribosomes both structurally and dimensionally more closely resemble ribosomes found in modern day bacteria, rather than those in eukaryotic cells. Mitoribosomes, however, display a 2:1 protein-to-rRNA mass ratio, much higher, than the 1:2 ratio in bacterial ribosomes. Mitoribosomes also replaced large parts of their non-core rRNA components with proteins as part of a devolutionary process, similar to observations of other symbionts and parasites [12]. A close examination of the mitoribosome structure reveals that the new proteinaceous component replaced rRNA around its periphery, creating an outer “shell” around the conserved catalytic rRNA core [13]. This suggests that these ribosomes developed a kind of “armor” to protect their catalytic rRNA cores from attack by reactive oxygen species, which are produced in abundance by the process of oxidative phosphorylation in mitochondria. Thus, we suggest that mitoribosomes may have become “specialized” to function in this particularly harsh environment.

Chloroplasts are also endosymbionts with their own genomes (plastome) and bacteria-like 70S ribosomes. While the general protein-to-rRNA mass ratio of chlororibosomes does not differ much from those in bacteria, they do contain unique rRNA features, five chloroplast-specific ribosomal proteins, and unique protein extension elements. These enable the specialized function of chlororibosomes by promoting the association of translation factors involved in light- and temperature-dependent control of plant protein synthesis [14,15]. These too may be considered to have become “specialized” to optimize protein expression in the unique environment of the chloroplast.

### 2.2. Extreme Ribosomes

The first atomic resolution structures were generated using ribosomes that are specialized to function in extreme environments [16,17,18]. The organisms from which they were purified, *Thermus thermophilis* and *Haloarculum marismortui*, evolved to thrive in environments of high temperature and in high osmolarity, respectively. Biochemically, such conditions tend to destabilize non-covalent interactions, particularly hydrogen bonds and salt bridges. Accordingly, the biomolecules synthesized by extremophiles have evolved to maximize stability, traits which make them ideal for crystallization studies. Thus, one may consider these ribosomes optimized to ideally function in their respective extreme environments. Given the ancient origins of the ribosome and its central role in biology, the idea that ribosomes can become environmentally specialized has profound implications for the field of astrobiology.

### 2.3. Ribosomes of Parasites

Parasites tend to be minimalists because a) their host organisms are able to meet most of the metabolic needs, and b) their requirements for small genomes that can be rapidly replicated. Their ribosomes also tend to follow the trend towards minimalization. For example, microsporidia are eukaryotic parasites that have successfully adapted to parasitize almost all animals. Their genomes have condensed to be the smallest known in the *Eukaryota*, and their mitochondria are rudimentary. A recent cryo-EM analysis of *Vairimorpha necatrix* revealed the smallest known eukaryotic cytoplasmic ribosome to date [19]. The rRNA from this species has been reduced to a functionally conserved core due to the loss or severe compaction of all of the eukaryote-specific expansion segments, and it lacks two eukaryote-specific ribosomal proteins, eL38 and eL41. Furthermore, this species lacks the 5.8 rRNA, whose core sequences have been fused with the large subunit rRNA to create a unique 23S rRNA species. Interestingly, these ribosomes also associate with MDF1 and MDF2, distinct dormancy factors that may allow these organisms to save energy by storing inactive, “hibernating” ribosomes when they are not needed for active protein synthesis, e.g., during the spore stage.

Trypanosomes comprise a genus of parasitic flagellated protozoa in the class Kinetoplastea, best known for causing a variety of infectious diseases, including sleeping sickness, cutaneous leishmaniasis, and Chagas disease. Unlike the microsporidia, which have minimized rRNA content, trypanosomal rRNAs have become enlarged, containing unusually large expansion segments, a large rRNA domain that is not found in other eukaryotes, and additional rRNA insertions [20]. Additionally, some of the ribosomal proteins contain unique extensions, which enable the formation of four inter-subunit bridges that are not observed in other eukaryotic ribosomes. Curiously, although the genomic rDNA genes encode the four rRNA species, the large subunit rRNA is cleaved into six unique pieces. The functional aspects of these unique features are currently unknown.

The Apicomplexia include the genus *Plasmodia*, best known as the parasites responsible for malaria. Interestingly, *Plasmodium* species carry two cytoplasmic ribosome variants with different rRNA compositions. One of these is expressed in the mosquito vector, and the other is present in the mammalian host, although both can simultaneously occur for limited periods of time [21]. Presumably, these maximize the ability of the organism’s ribosomes to function in the very different environments of the insect vector and human host.

## 3. Aliens Within Us

In contrast to the ideas presented in the previous section, the concept of “specialized” ribosomes is more popularly associated with otherwise normal cytoplasmic ribosomes that, by virtue of a change in protein or rRNA content, are endowed special properties. The reader is directed to reviews by the Barna [8] and Blanchard research groups [22], whose research efforts deeply explore this topic of inquiry in mammalian and bacterial cells, respectively. Here, we explore emergent and perhaps more controversial examples of this type of specialization.

### 3.1. Ribosomal Protein Paralogs

Mammalian ribosomes harbor 80 ribosomal proteins. However, the existence of splice variants and paralogous versions of some of these suggests that incorporating one in favor of another may confer some specialized function (reviewed in [23]). Of particular interest are instances of ribosomal proteins that originally arose from gene duplication and are actively expressed, but which also have variations in their amino acid sequences. These include three versions of the *RPS4* gene (*RPS4X* on the human X-chromosome, and *RPS4Y1&2*, both residing on the Y-chromosome), and genes encoding “ribosomal protein-like” proteins (*RPL3L*, *RPL7L, RPL10L, RPL22L*, *RPL24D1L*, *RPL26L*, *RPS27L, RPL36aL,* and *RPL39L*). All of these are actively transcribed. *RPL7L* and *RPL24D1* are currently thought to be exclusively involved in ribosome assembly and not incorporated into actively translating ribosomes. However, the hypothesis that one or both of these may be incorporated into mature ribosomes devoted to some special purpose remains to be disproved. Perhaps the strongest evidence for specialization comes from work on *RPL3L*, which appears to be exclusively expressed in striated muscle tissue [24]. Hypertrophic stimulus of skeletal muscle inhibited Rpl3lp expression to 20% of baseline levels, while increasing the expression of Rpl3p approximately five-fold, suggesting that Rpl3lp functions as a negative regulator of muscle growth. This is supported by the observation that induction of Rpl3lp expression in myoblasts during myotube formation greatly impaired myotube growth myoblast fusion. Furthermore, muscle growth requires active ribosome biogenesis [25]. Given the key role of ribosomal protein L3 in ribosome biogenesis and function [26], we suggest that mature muscles may utilize ribosomes harboring Rpl3lp instead of Rpl3p to retain homeostasis in mass. Stimuli that lead to muscle growth, such as injury and exercise, may cause striated muscle cells to switch to ribosomes harboring Rpl3p.

### 3.2. Immunoribosomes

The plasma membranes of nucleated cells in jawed vertebrates present Major Histocompatability Complex (MHC) Class I proteins in combination with antigenic peptides. These peptides are proteolytic products derived from cellular proteins. It has been proposed that defective ribosomal products (DRiPs) are a major source of these antigenic peptides [27]. The linkage between protein synthesis and MHC Class I antigen production prompted the proposal of a special class of ribosomes, the “immunoribosome”, which is specifically dedicated to producing DRiPs [28]. While still controversial, a substantial body of evidence has emerged supporting this immunoribosome hypothesis over the past decade (reviewed in [29]). Going forward, the challenge will be to demonstrate the presence of such dedicated ribosomes using biochemical and/or genetic approaches. Success in this endeavor may have far-reaching consequences on our understanding of how homeostasis at the cellular level may be linked to immune surveillance at the organismal scale.

### 3.3. Onco-Ribosomes

In addition to the well-described ribosomal mutations in the congenital ribosomopathies, mutations in several ribosomal proteins in somatically acquired cancers have recently been described (reviewed in [30,31,32]). For example, alterations in RPs such as RPL5 (uL5), RPL10 (uL16), RPS15 (uS19), RPL11 (uL15), and RPL22 (eL22) have been described in 10–40% of multiple tumor types. Studies of some of these suggest that, in addition to negatively impacting ribosome assembly similarly to RP mutations in ribosomopathies, the somatic RP mutations also influence ribosomal function, resulting in an oncogenic rewiring of the cellular protein expression profile. A well-studied example is the *RPL10–R98S* mutation in T cell leukemia, which promotes specific overexpression of the oncogenic JAK–STAT signaling cascade [33], IRES-dependent overexpression of the anti-apoptotic factor BCL2 [34], and both transcriptional and translational upregulation of serine and glycine biosynthesis [35]. Other examples include elevated expression of the oncogene c-MYC upon *RPL11* inactivation [36], induction of stemness factor Lin28B by *RPL22* inactivation [37], and altered translation of the critical hematopoietic transcription factor GATA1 in the RP-mutant ribosomopathy Diamond Blackfan anemia, which is in turn associated with a high risk of cancer progression [30,38]. More generally, ribosomal protein lesions have recently been described to promote cellular oxidative stress and increased mutagenesis [39].

Aside from mutations in RPs, rRNA modifications such as methylation and pseudouridylation are frequently altered in cancer cells [40]. It is tempting to speculate that unique modification patterns could also translate into unique gene expression patterns. In support of this, disruption of dyskerin, which catalyzes pseudouridylation, or of small nucleolar RNAs (snoRNAs) that guide dyskerin to rRNA is found in many cancers and can impair the translation of tumor suppressor-encoding mRNAs [40]. While it is too early to definitively proclaim the existence of an onco-ribosome, the recent studies supporting a specialized function of ribosomes in cancer underscore that ribosomal diversity can play a key role in human disease.

## 4. The Expanding Universe of Ribosome Diversity

**Figure 1:**
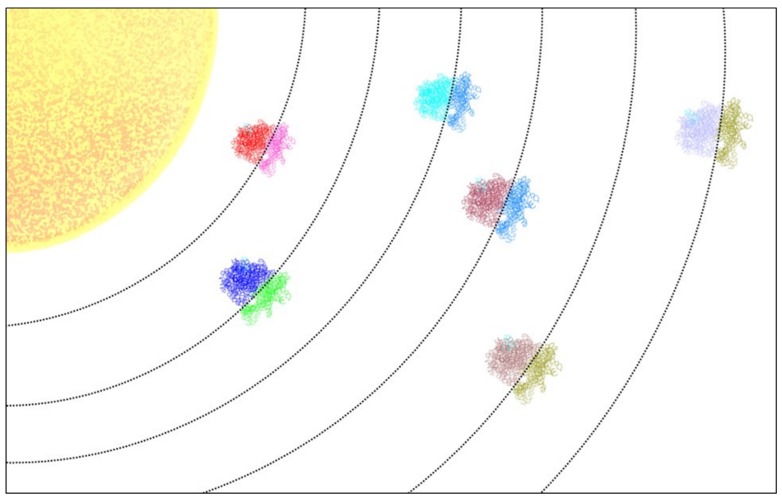
The ribo-system. Various types of ribosomes populate the known riboverse. Each type of ribosome is specialized for particular environments, which are represented by orbits in the above image. Each orbit corresponds to each ribosomal species’ “habitable zone”.

The number of known exoplanets is now in the thousands. Taking into account the possible variations in parameters such as mass, orbit, composition, and distance to its star or stars, the number of conceivable unique planets approaches infinity. In parallel, the large number of known and possible ribosomes might be thought of as a constellation of ribosomes, which we call the “ribo-system” (Figure 1). 

The rate of expansion in our knowledge of the degree of heterogeneity among ribosomes is similarly expanding, representing an exciting field of research. In addition to the different ribosomal protein paralogs discussed above, the functional importance of differences in their post-translational modification is beginning to emerge.

Recent advances, particularly in single-cell sequencing and quantitative mass spectroscopy, are helping to bring the visible riboverse into sharper focus. In mammals, the observation of tissue-specific patterning defects in mice lacking the *RPL38* gene and its linkage to defects in translation of specific homebox (HOX) mRNAs represented a seminal breakthrough because of its implications for ribosome specificity in developmental biology [41]. The demonstration of the importance of this protein for translation of IRES-containing mRNAs created a new paradigm with regard to ribosome-mediated control of gene expression [9]. However, the idea of generating heterogeneity through subtraction [42] is less appealing than showing specificity through substitution of one ribosomal protein variant for another, e.g., the Rpl3l case described above. Later, sophisticated proteomics analyses revealed non-stoichiometric levels of ribosomal proteins, their association with various classes of other proteins, and association with different transcript sub-pools, painting a picture of ribosomes specialized by the ability of intrinsic protein content to recruit specific *trans*-acting factors [43,44]. Similarly, the evolution of rRNA expansion segments has been found to provide new platforms for binding *trans*-acting factors required for recruitment of specific mRNA classes [45].

Ufmylation is a metazoan-specific post-translational modification in which UFM1 proteins are conjugated to particular ribosomal proteins. The finding that differences in ufmylation confers differences in specificity for *trans*-acting proteins suggests another route for specialization via differences in post-translational modification of ribosomal proteins [43]. Since then, evidence for such has steadily accumulated [46,47,48,49,50,51,52,53,54,55,56]. Post-transcriptional modification of ribosomal rRNAs presents a similar path to ribosome specialization [57,58]. In particular, the recent findings of variably methylated rRNA bases [59] and of changes in rRNA modification levels in response to external stimuli [60] suggest another avenue through which raised ribosome function and specificity may be regulated.

Ribosome specialization can also be achieved through allelic variation, the most well-documented example of which comes from recent studies of rRNA. For example, the *E. coli* genome contains seven distinct rRNA operons, each with their specific sequence variants [61]. RNAseq analyses were used to detect differences in the utilization of specific rRNA operons in response to nutrient limitation-induced stress, and these correlated with changes in ribosome function, gene expression, and cellular physiology, thus demonstrating specific roles for rRNA allelic variants [62]. In eukaryotes, rDNA copy number varies widely, and a cursory analysis of the human and mouse rDNA sequences revealed the potential for sequence heterogeneity within rDNA operons [63]. More recently, a meta-analysis of human and mouse genome databases identified pervasive intra- and inter-individual nucleotide variation in the 5S, 5.8S, 18S, and 28S ribosomal RNA (rRNA) genes of both human and mouse, and ribosomes bearing variant rRNA alleles were found to be present in the actively translating ribosome pool [64]. These findings strengthen the idea that physically and functionally heterogeneous ribosomes may be important for normal physiological development and homeostasis and, conversely, in pathological processes. Allelic variation may also play an important role in population biology and evolution. This may provide a means though which a species could be pre-adapted to survive fluctuations in environmental conditions, e.g., climate change or location-specific differences in micronutrient availability.

## 5. Conclusions and Perspectives

### 5.1. Medical Implications of the Riboverse

Mirroring the inappropriate moniker “The Ribosome”, we frequently refer to “cancer” as if it were one disease. However, to borrow from a Buddhist expression—there are many paths to enlightenment. In the cancer context, this refers to the path towards transformation, and almost no two cells follow the exact same path. Cancer thus ultimately encompasses many diseases. And heterogeneity is a hallmark of cancer at every level, as even one patient’s tumor often displays several mutant clones, and still, within one clone, inter-cellular variability. To therapeutically overcome the tremendous hurdle posed by cancer’s heterogeneity, it is essential to continuously improve the technologies illuminating the altered wiring of cancer cells at the DNA, RNA, and protein levels.

While proteins are the chief cellular performers, tumor protein biology has been largely inaccessible and under-explored, as proteomics technologies have lagged for several decades behind other -omics technologies. The flow from DNA to RNA to protein is accompanied by an exponential increase in complexity, and this entire layer of additional potential variation within tumors has thus largely remained out of view. The cancer genome atlas (TCGA) contains full transcriptome, exome, and genome data for thousands of tumor samples, but the first full quantitative mass spectrometry-based proteomic descriptions of these cancer datasets are only now appearing (e.g., [65]). We have just begun to build correlations between tumor genomes, transcriptomes, and proteomes, giving rise to terminology such as “proteogenomics”. Such initial analysis enabled the identification of significant RNA–protein discordances, indicative of translational dysregulation. There is now a plethora of examples in both congenital and somatic ribosomopathies that support the differential translational output of specialized onco-ribosomes stemming from RP or ribosome biogenesis factor defects (reviewed in [32]). This provides an additional facet to the vast heterogeneity of cancer, but one that is beginning to be therapeutically exploited.

Over half of existing antibiotics bind and inhibit prokaryotic ribosomes. The selectivity of antibiotic binding is often provided by very subtle differences in ribosomal structure, and resistance to antibiotics is often enabled by changes as minor as a single chemical modification or the position of a single nucleotide in the ribosome [66,67]. Unique species-specific ribosomal features are being continuously discovered. For example, as noted above, the 80S ribosome from the malarial *P. falciparium* harbors parasite-specific structural elements. These are currently being explored as targets for the rational design of small molecules to specifically inhibit the parasitic translational apparatus [68]. It should therefore be feasible to also develop small molecules that target RP-mutant cancer ribosomes, provided that these have distinguishing structural features. However, the exact composition of these specialized ribosomes has not been elucidated. In-depth structural studies by cryogenic electron microscopy or X-ray crystallography, combined with analysis of the protein composition, the rRNA modification status, and the spectrum of interacting proteins (ribo-interactome), would be required to characterize these specialized ribosomes in more detail. This might in turn encourage the development of “specialized” translation inhibitors. Indeed, new classes of translation inhibitors are being developed that selectively target translation of small subsets of mRNAs, with few off-target effects [69,70]. Illuminating the structure and function of oncogenic ribosomes, along with discovering their inhibitors, could enable a novel promising type of personalized therapy.

Such an approach might also find applications with regard to mitochondrial ribosomes, as a growing body of evidence suggests an important role for mitoribosomes in cancer progression. Cancer cells generally display higher rates of mitochondrial biogenesis [71] and their fitness is improved through a concerted increase in both cytosolic and mitochondrial translation [72]. Moreover, mitochondrial translation inhibition was proposed as an attractive therapeutic strategy for acute myeloid leukemia [71]. The clinical relevance of mitochondrial translation is further highlighted by the occurrence of mutations in mitochondrial RP genes, associated with mitochondrial dysfunction disorders (reviewed in [12]). For example, a recent case report highlights a common familial mitochondrial mutation that sensitizes the affected individuals to the ototoxicity of aminoglycoside antibiotics due to the mutation-induced structural changes in the mitoribosome [73]. While somatic mutations in mito-RP genes also regularly appear in cancer genomics datasets, their significance in cancer pathogenesis is not yet fully understood.

Finally, ribosome heterogeneity might play a causative role in another class of ribosome-mutant disorders: Ribosomopathies. These disorders stem from ribosome dysfunction and display a broad spectrum of phenotypic defects. However, most of these diseases also display similar hematopoietic deficiencies, such as bone marrow failure and anemia. This intriguing tissue specificity paradox begs the question of why mutations in the biochemical machine found in every single cell of the body, and with an essential role in every tissue, tend to have a more profound effect on hematopoietic cells. The notion that ribosomes can vary in composition between different tissues, as discussed above, may shed some light on this paradox. For example, a quarter of human ribosomal proteins exhibit tissue-specific expression, and primary hematopoietic cells display the most complex expression patterns [74]. Additionally, a recent study indicates that ribosomal proteins found mutated in ribosomopathies like Diamond Blackfan Anemia are substoichiometric and demarcate ribosomes with specialized functions [44]. This study demonstrated how haploinsufficiencies of specific ribosomal proteins particularly perturb the translation of specific mRNAs, which may disproportionately result in hematopoietic dysfunction. Moreover, while the precise roles of the recently discovered ufmylation, discussed above, remain to be determined, the available knockout mouse models for the enzymes of the ufmylation cascade show defects in erythrocyte differentiation and result in embryonic lethality [75]. This is highly similar to the defects arising from haploinsufficiency of some ribosomal proteins in several ribosomopathies. These initial studies suggest that the specialized composition of the translational machinery in hematopoietic tissues might make them more vulnerable to defects in ribosomal function and regulation.

### 5.2. Designer Ribosomes

Some of the first yeast molecular genetics studies involved screening for resistance to antibiotics. One of these, the peptidyltransferase inhibitor trichodermin, was used to clone the *TCM1* gene [76]. Contemporaneously, a genetic screen for mutants unable to maintain the yeast killer virus identified a family of *MAK* (MAintenance of Killer) genes, including *MAK8* [77]. A subsequent study demonstrated that both the *TCM1* and *MAK8* encoded ribosomal protein uL3 (RPL3) and that they both carried the W255C mutation [78], which was later shown to structurally alter the peptidyltransferase center, thus accounting for its ability to confer resistance to a broad range of peptidylteransferase inhibitors [79]. Importantly, this was the first demonstration that ribosomes could be manipulated to incorporate novel functions, i.e., antibiotic resistance. The list of human-made mutations in ribosomal proteins, in both bacteria and yeast, is long and growing [80]. rRNA is equally amenable to bioengineering approaches, including insertion of aptamers, which can be used to facilitate affinity-based applications [81]. More recently, both *E. coli* and *S. cerevisiae* ribosomes have been bioengineered to contain a single rRNA, greatly facilitating the creation of “designer” ribosomes [82,83]. Given the malleability of ribosomes and the expanding set of tools that can be used to create new variations, the theoretical possibilities are manifest. For example, ribosomes may be created to optimize synthesis of therapeutic proteins containing novel amino acids. They could be engineered to polymerize novel functionalized monomers or perhaps nanostrings having incredible strength or utility for their informational complexity. Indeed, it is not pure science fiction to contemplate creating ribosomes designed to function in microorganisms with a particular purpose, and we dare to speculate that such organisms may play roles in the terraforming of Mars, Europa, or Enceladus in the not-so-distant future.

## 6. Final Remarks: Looking Backwards and Forwards

**Figure 2:**
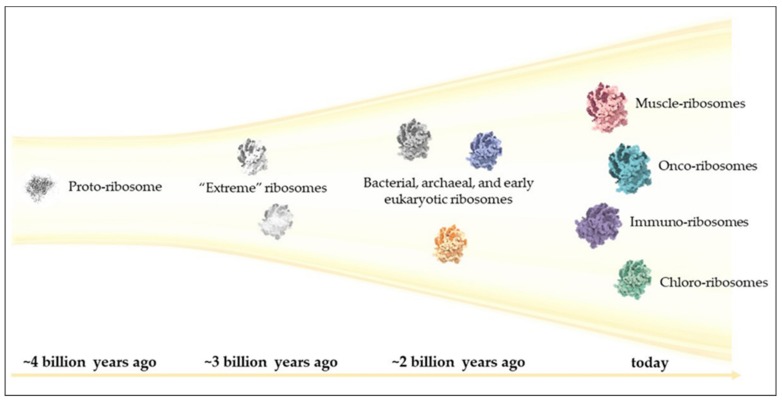
The expanding riboverse. The primordial ribosomal core, the proto-ribosome, is thought to have evolved approximately 4 billion years ago, marking the “Big Bang” of the riboverse. This ancient molecule continued to structurally and functionally evolve along with cellular evolution, diversifying and specializing to thrive in an increasing number of environments. Recent evidence suggests that in addition to inter-species specialization, ribosomal diversity also exists at the intra-organismal level.

The potential number of unique ribosomes is on the same order of magnitude as the number of stars in the visible universe [63], and like our universe, it is constantly expanding (Figure 2). Nevertheless, direct evidence for specialized ribosomes remains elusive, and failure to uncover such evidence is more prevalent than success [84]. Extraordinary claims require extraordinary evidence; thus, the criteria for establishing the existence of specialized ribosomes must be stringently defined. Proposals include the biochemical “one enzyme, one substrate” approach [63], as well as genetic methodologies, e.g., demonstrations of gain-of-function mutation and inducibility under specific physiological conditions [85]. However, it is now clear that ribosomal heterogeneity abounds. The remarkable durability of life in the face of at least five mass extinction events during the Earth’s planetary history may be attributable to the remarkable degree of heterogeneity that can be tolerated by ribosomes. Given the challenges currently facing humankind, our future may, at least in part, depend on our ability to manipulate this central organelle.
